# Anti-high mobility group box-1 (HMGB1) antibody attenuates kidney damage following experimental crush injury and the possible role of the tumor necrosis factor-α and c-Jun N-terminal kinase pathway

**DOI:** 10.1186/s13018-017-0614-z

**Published:** 2017-07-12

**Authors:** Bin-Fei Zhang, Peng-Fei Wang, Yu-Xuan Cong, Jin-Lai Lei, Hu Wang, Hai Huang, Shuang Han, Yan Zhuang

**Affiliations:** Department of Orthopedic trauma, Honghui Hospital, College of Medicine, Xi’an Jiaotong University, Beilin District, No. 555 Youyi East Road, 710054 Xi’an, Shaanxi Province People’s Republic of China

**Keywords:** High mobility group box-1, Crush injury, Tumor necrosis factor-α (TNF-α), c-Jun N-terminal kinase (JNK), SP600125, Apoptosis

## Abstract

**Background:**

Inflammation plays a crucial role in kidney damage after crush syndrome (CS). Several researchers report that high mobility group box-1 protein (HMGB1) may be the vital trigger in kidney damage, and tumor necrosis factor-α (TNF-α) and c-Jun N-terminal kinase (JNK) are involve in this pathophysiological process, but their biological roles are unclear. This study aimed to explore the relationship between HMGB1, JNK, and TNF-α in kidney damage.

**Methods:**

The crush injury model was established using weight compression. The reliability of the crush injury model was determined by hematoxylin-eosin (HE) staining. Western blot was used to detect the expression of HMGB1, JNK, and TNF-α, and TUNEL was used to mark apoptotic cells in the renal cortex.

**Results:**

The results showed that the highest expression of HMGB1 in muscle was 12 h after CS. JNK and TNF-α increased and peaked at 1 day after CS in kidneys. Western blot analysis revealed that anti-HMGB1 antibody could downregulate the expression of JNK and TNF-α. Anti-TNF-α could downregulate activation of JNK, and SP600125 could downregulate expression of TNF-α in the kidneys. In addition, anti-HMGB1 antibody, anti-TNF-α antibody, and SP600125 could reduce cellular apoptosis in the renal cortex.

**Conclusions:**

It is possible that JNK and TNF-α commonly contribute to kidney damage by assembling a positive feedback cycle after CS, leading to increased apoptosis in the renal cortex. HMGB1 from the muscle may be the trigger.

## Background

Crush syndrome (CS) is a serious medical condition that can develop after traumatic events, such as earthquakes, landslides, and vehicle accidents [1]. Most studies have highlighted symptoms of circulatory shock, renal failure, and cardiac arrhythmia [1–3] and focuses on early fluid resuscitation, forced diuresis, and renal replacement therapy as treatments [3–5]. Even though these supportive treatments are carried out, patients often present systemic inflammatory response syndrome or fall into multiple organ failure, leading to death in the following stage, such as distant lung injury [6, 7] and cardiomyocyte-specific injury [8] post-CS. Thus, the influence of crush injury is widespread, and the knowledge of it is currently limited.

Recently, numerous studies have focused on high-mobility group box 1 protein (HMGB1), since HMGB1 is reported to have a crucial role in the pathogenesis of systemic acute inflammation [9, 10]. In CS, the level of serum HMGB1 has been found to peak at 3 h after releasing compression [7]. It has additionally been found that administration of anti-HMGB1 antibody improves survival rate and suppresses serum levels of HMGB1 and inflammation, and could therefore ameliorate lung damage [7]. Therefore, HMGB1 has become a key therapeutic target. As for kidney damage after CS, excluding the role of myoglobin to renal tubular obstruction directly [11], HMGB1 also may contribute to the damage. Although the detailed mechanisms of kidney damage are not clear, clinical and experimental studies indicate that inflammatory mediators are associated with advanced kidney damage [7, 12].

Previous studies have demonstrated that HMGB1 could increase the production of TNF-α indirectly [13] and also increase the level of c-Jun N-terminal kinase (JNK), known as stress-activated protein kinase [14]. TNF-α is an important inflammatory mediator produced by many cells and tissues [15]. TNF-α has a direct role in kidney damage [16] and can also induce apoptosis and necrosis in various cells [17, 18], including renal tubular apoptosis [19]. Neutralization of TNF-α has been found to reduce renal fibrosis with renal failure [20]. JNK could also produce inflammatory mediators, especially TNF-α [21]. Additionally, JNK is also one of the pathways activated by TNF-α [22]. Thus, a positive feedback cycle involving JNK and TNF-α seems to exist, substantively contributing to cell damage in the kidneys.

However, the trigger effect of HMGB1 and the relationship between JNK and TNF-α expression is uncertain. In addition, it is not clear whether administration of anti-TNF-α, anti-HMGB1 antibodies, and SP600125, a JNK inhibitor, exerts a protective effect against TNF-α induced kidney damage. We investigated whether these administrations affected the apoptosis in kidneys.

## Methods

### Experimental model of CS

In total, 90 C57BL/6 male mice weighing 20–25 g were purchased from the Laboratory Animal Center of Xi’an Jiaotong University. Animals were housed and fed in a temperature- and humidity-controlled environment with standardized light/dark cycle (12 h day/night) for 1 week. All animal procedures were in accordance with the ARRIVE guidelines and the National Institutes of Health guide for the care and use of Laboratory animals. The study was approved by the Ethics Committee of Xi’an Jiaotong University.

In the first phase, 30 mice were randomly assigned to 2 groups: normal group (*n* = 6), and CS group (*n* = 24). Animals in the CS model were anesthetized with an intraperitoneal injection of 10% chloralhydrate (0.35 mL/kg) and were placed in a prone position, with both hind limbs (2 cm from the ankles up) compressed by 20 kg weights [23]. The 6-h period of compression was selected because the majority of CS victims are entrapped under the rubble between 5 and 8 h [24]. Body temperature was maintained at 37 ± 0.5 °C during the operation. Euthanasia was carried at 0 h, 12 h, 1 day, and 2 days (*n* = 6 in each group) after compression. Mice in the normal group were euthanized on day 2. One kidney and muscle from one hind limb in each mouse was taken for HE staining, and the remaining muscle and kidney were obtained for western blotting.

### Drug administration

In the second phase, 60 mice were randomly assigned to 5 groups: CS (*n* = 12), CS + vehicle (*n* = 12), CS + anti-HMGB1 antibody (*n* = 12), CS + anti-TNF-α antibody (*n* = 12), and CS + SP600125 (*n* = 12). The CS model was established as described above. Intraperitoneal injections were performed 1 h after compression was released, at a total volume of 200 μl. Dimethyl sulfoxide (DMSO) was used as a solubilizer. The dosage of anti-TNF-α antibody (5 μg/200 μl), SP600125 (10 μg/200 μl), and anti-HMGB1 antibody (2.5 μg/200 μl) [25] was based on previous studies. In the vehicle group, only the same amount of DMSO was injected. Euthanasia was performed according with the results in phase 1. All mice were sacrificed for western blotting or TUNEL staining.

### HE staining

HE staining was performed to observe the pathological changes in muscle and kidney. The pathological tissues were fixed in normalized fixative, consisting of 4% paraformaldehyde in 0.01 M phosphate-buffered saline, overnight at room temperature. The tissue blocks were then dehydrated with an ascending ethanol series, cleared with xylene and then embedded in paraffin. The paraffin blocks were cut into transverse serial sections of 10 μm thickness. Next, five sections including skeletal muscle and kidney from each animal were randomly chosen and mounted on poly-L-lysine coated slides for HE staining.

### Western blot analysis

The frozen tissue samples were solubilized in RIPA buffer on ice using a homogenizer. Samples (50 μg/lane) were separated on a sodium dodecyl sulfate-polyacrylamide gel 12% gel) and electrotransferred onto polyvinylidene fluoride membranes. After 2 h incubation in blocking solution (5% non-fat milk in 20 mM Tris-HCl, 150 mM NaCl, 0.1% Tween-20; TBST), the membranes were blotted with primary antibodies against HMGB1 (1:1000, CST), TNF-α (1:1000, CST), p-JNK (1:1000, CST), JNK (1:1000, CST), or β-actin (1:1000, Beijing Biosynthesis) overnight at 4 °C. After extensive rinsing with TBST buffer, the blots were incubated with HRP-conjugated anti-rat/mouse secondary antibodies (Abgent, San Diego, CA, USA). Specific bands were detected using X-ray film. The density of the bands was analyzed by Quantity one software version 4.62 (Bio-Rad, USA).

### TUNEL staining

We used a terminal deoxynucleotidyl transferase-mediated dUTP nick end-labeling (TUNEL) reaction by means of TUNEL Kit (Roche, Germany) to detect apoptotic cells. The tissue sections were deparaffinized with xylene, and were treated with H_2_O_2_ in methanol to inactivate endogenous peroxidase. After washing with PBS, specimens were incubated in the labeling reaction mixture containing terminal deoxynucleotidyl transferase. After incubation, the sections were incubated with horseradish peroxidase. The sections were then treated with DAB solution and were counterstained with hematoxylin. Finally, the sections were mounted to a glass slide with a cover slip.

### Statistical analysis

SPSS 18.0 (SPSS Inc., Chicago, IL, USA) was used for statistical analyses. All data were presented as mean ± SD. Comparisons among multiple groups were performed using ANOVA. Comparisons between the two groups were performed using the LSD test. *P* < 0.05 was considered statistically significant.

## Results

### Mortality rate and general observations

In total, 25 mice died after the operation and administration of drugs during the experiments. There was no death in the normal, while the number of deaths was 1, 3, 2, and 2 in the 0 h, 12 days, 1 day, and 2 days groups, respectively. The death in 0 h was during the process of compression. In the second phase, 5, 5, 2, 3, and 2 deaths were observed in the CS, CS + vehicle, CS + anti-TNF-α antibody, CS + SP600125, and CS + anti-HMGB1 antibody groups, respectively. The 1 and 2 deaths in CS and CS + vehicle were during the process of compression. After CS, physical activities of mice reduced obviously, with both hind limbs prolapsing. Dead mice were excluded from further analysis. In all the living mice, injured skeletal muscles were found to be hemorrhagic and with edema.

### Pathological changes in muscles and kidneys after CS

**Fig. 1:**
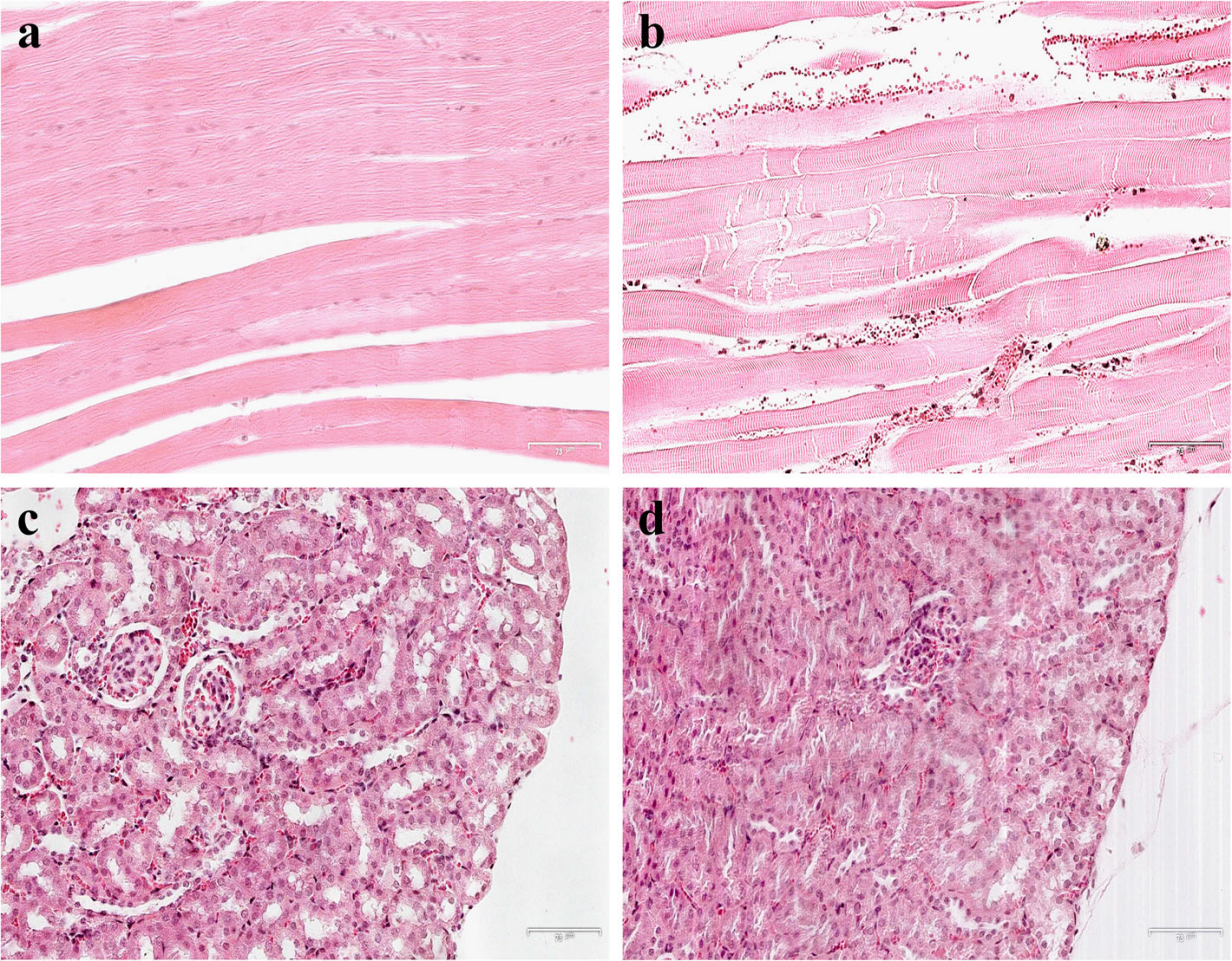
HE staining in the muscle and kidney (×200). There are 6, 4 mice in the normal (**a**, **c**) and CS 1d (**b**, **d**) group, respectively

Pathological changes in muscles and kidneys were detected via HE staining, to assess the reliability of our CS model. As shown in muscle pathological sections, there was bleeding, degeneration, swelling, and necrosis apparent in part of the muscle fibers and lymphocytic infiltration in the CS groups (Fig. 1b), compared to the normal tissue (Fig. 1a). In the kidneys of CS groups, part of the renal capsule thickened, epithelial cells swelled, and some showed vacuolar degeneration or necrosis. Necrotic cell debris and exudates were found in part of the renal tubular lumen, and myoglobin casts observed (Fig. 1d).

### Dynamic changes of HMGB1, JNK, and TNF-α in muscles and kidneys

**Fig. 2:**
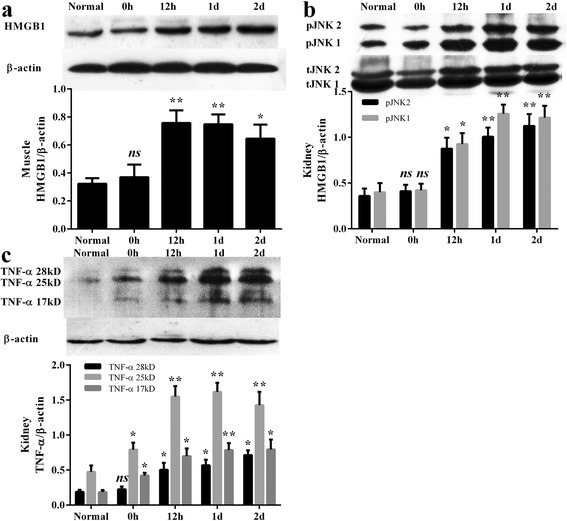
Representative western blot and quantitative analysis of HMGB1, p-JNK, t-JNK, TNF-α, and β-actin in the muscle (**a**) and kidney (**b**, **c**). There are 6, 5, 3, 4, 4 mice in normal, 0 h, 12 h, 1 day, and 2 days group, respectively. Results are described as mean ± SD. **p < 0.01, *p < 0.05, ns *p* > 0.05 versus the normal group

Western blot analysis revealed a significant upregulation of HMGB1 in muscle, and p-JNK, and TNF-α in kidneys following CS, compared with the normal groups. It showed a gradual increase of HMGB1 expression until reaching a peak 12 h after crush injury in muscle tissue (Fig. 2a). In kidneys, p-JNK/t-JNK also showed an increasing trend, with p-JNK2 and p-JNK1 peaking 1 day after injury (Fig. 2b). Similarly, the level of three types of TNF-α in the CS group was also higher than in the normal groups, peaking at 1 day (Fig. 2c) in the kidneys.

### Anti-HMGB1 antibody effect on JNK and TNF-α expression in the kidneys

**Fig. 3:**
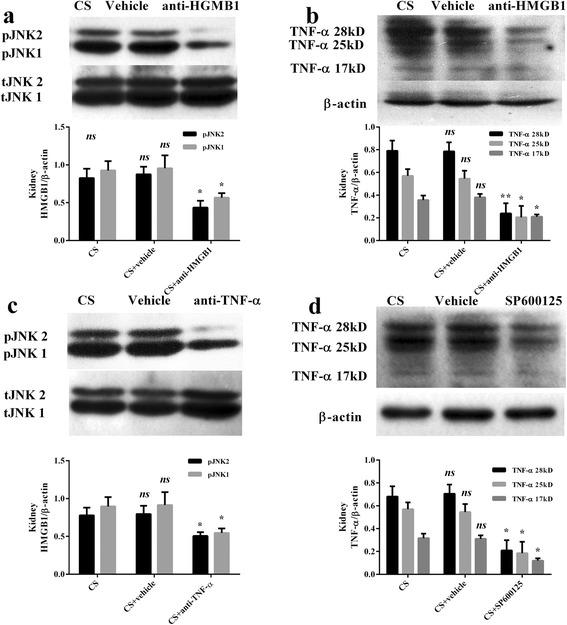
Representative western blot and quantitative analysis of p-JNK, t-JNK, TNF-α, and β-actin in the kidney. There are 7, 7, 10 mice in CS, CS + vehicle, and CS + anti-HMGB1 antibody group, respectively (**a**, **b**). There are 7, 7, 10, 9 mice in the CS, CS + vehicle, CS + anti-TNF-α antibody, CS + SP600125 group, respectively (**c**, **d**). Results are described as mean ± SD. **p < 0.01, *p < 0.05, ns *p* > 0.05 versus the normal group.

Since the expressions of JNK and TNF-α increased to a peak 1 day post-CS, we chose this time point to sacrifice mice for the second phase experiments. When anti-HMGB1 antibody was administered to block the activation of p-JNK and production of TNF-α, western blot analysis revealed that anti-HMGB1 induced a downregulation of p-JNK and TNF-α expression, compared with the high levels detected in the CS and vehicle group (Fig. 3a, b).

### Anti-TNF-α antibody effect on JNK and SP600125 effect on TNF-α expression in the kidneys

When anti-TNF-α antibody was administered, western blot analysis showed that p-JNK/t-JNK was downregulated compared to the CS group (Fig. 3c). When SP600125 was administered, western blot analysis showed that TNF-α was also downregulated compared to the CS group (Fig. 3d).

### TUNEL staining in kidneys

**Fig. 4:**
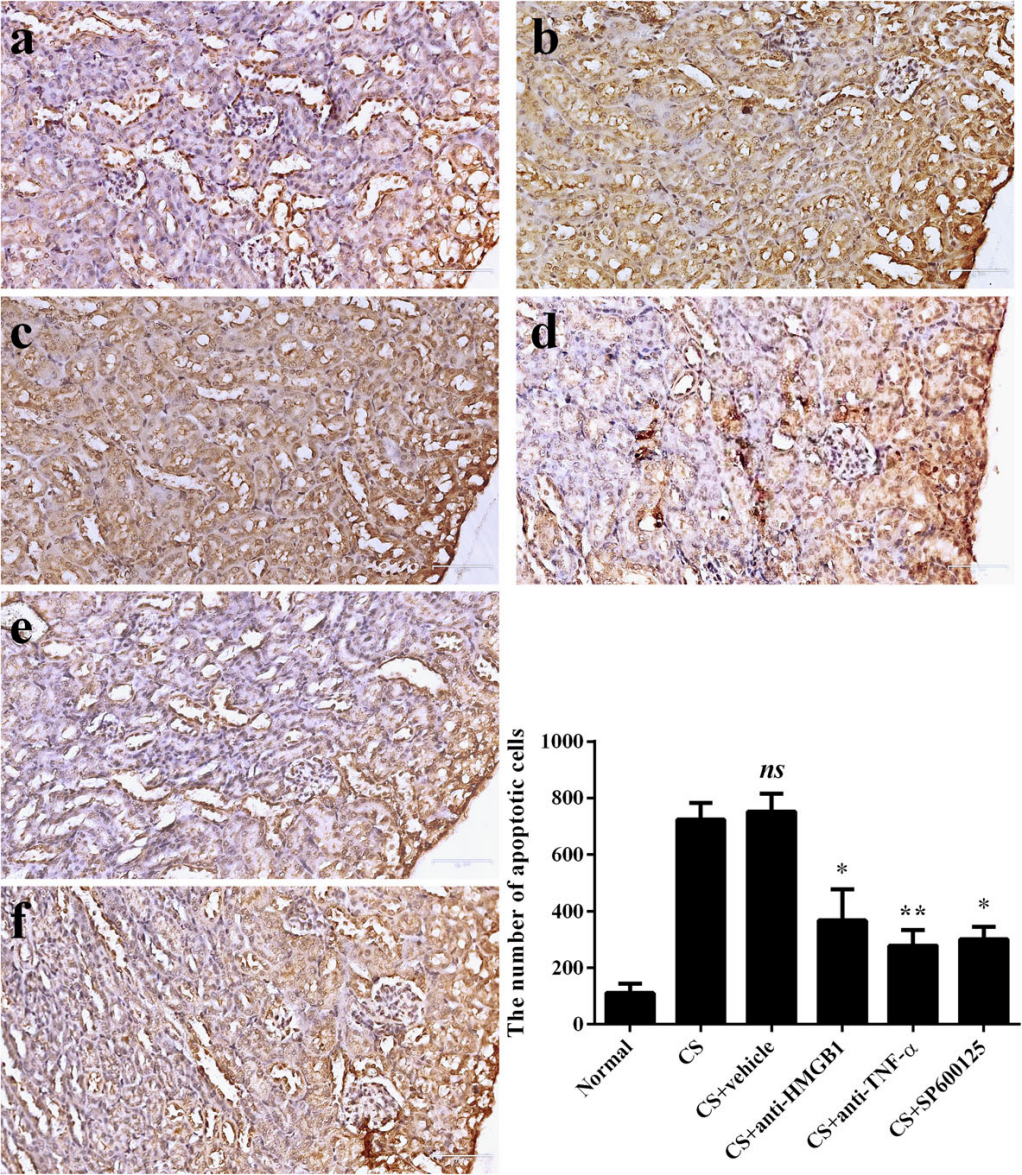
TUNEL staining label apoptotic cells in the renal cortex at 12 h after releasing (×200). Positive cells are dark brown. There are 6, 7, 7, 10, 10, 9 mice in the normal (**a**), CS (**b**), CS + vehicle (**c**), CS + anti-TNF-α antibody (**d**), CS + anti-HMGB1 antibody group (**e**), and CS + SP600125 (**f**), respectively. The number of positive cells was calculated in 5 fields in each section, and in 5 sections from each mouse. Results are described as mean ± SD. ** *p* < 0.01, * *p* < 0.05, ns *p* > 0.05 vs. the CS + vehicle group

There were few TUNEL-positive cells in the normal renal cortex region (Fig. 4a), but the number of positive cells increased sharply in CS (Fig. 4b), most of them tubular epithelial cells. There were no significant differences between the CS (Fig. 4b) and CS + vehicle groups (Fig. 4c). When anti-HMGB1 antibody was administered, results showed less observable apoptotic cells in the renal cortex (Fig. 4d). When anti-TNF-α antibody (Fig. 4e) or SP600125 were administered (Fig. 4f), analyses showed that they decreased the level of apoptosis in the cortex respectively, compared with the vehicle group.

## Discussion

This study focused on the trigger role of HMGB1 from the muscle, and the detailed relationship between JNK and TNF-α in the kidneys. Our results indicate the following main findings. Firstly, HMGB1 and pro-inflammatory cytokines are activated rapidly in the skeletal muscle and released from the nucleus to the extracellular matrix. JNK is then activated gradually in the kidneys, while TNF-α shows a similar tendency to JNK. Secondly, JNK and TNF-α in the kidney can be downregulated by blocking the action of HMGB1 (anti-HMGB1 antibody). Three types of TNF-α in kidney can be downregulated by blocking the action of JNK (SP600125). Thirdly, administration of anti-HMGB1, anti-TNF-α, and SP600125 can decrease apoptosis in kidneys, respectively.

Currently, the animal models of CS are mainly rats [26], mice [27], or rabbits [28], and modeling is derived from rubber tourniquets [26] or compression [6]. In this study, 20–25 g mice was compressed by weights to mimic acute injury and to create a condition that maximally resembled entrapment of the extremities under heavy rubble during a mass disaster. The 6 h period of compression was selected because most CS victims are entrapped under the rubble for between 5 and 8 h [24]. Histology was used to test the reliability of our CS model. There is swelling and degeneration of muscle fibers in the hind limbs and renal tubular obstruction in the kidneys, which indicate a successful CS model. To find the dynamic expression of HMGB1 in injured muscle and JNK and TNF-α in kidney, and to detect the regulation of these indicators, western blot was used. This study represents the first use of western blot to detect the expression of HMGB1, JNK, and TNF-α in muscle and kidney tissue after CS, as previous studies assayed using enzyme-linked immunosorbent technology.

Based on our results, HMGB1, JNK, and TNF-α seem to assemble a positive feedback cycle. HMGB1 from injured muscle activates JNK in the kidneys and JNK enhances production of TNF-α, which in turn enhances the production of JNK, resulting in soaring TNF-α level, which contributes to kidney damage. The level of HMGB1 in serum would be a meaning marker to evaluate the severity of CS injury. SP600125 and anti-HMGB1 antibody may be the possible drugs to alleviate the inflammatory response and damage.

Anti-HMGB1 antibody is reported to have a neutralizing effect on renal ischemia-reperfusion injury, and it also suppresses the number of apoptotic cells [25]. The results of this study show not only a reduction of apoptotic cells, but also a downregulation of JNK and TNF-α expression in kidney. Simultaneously, SP600125, an inhibitor of JNK [29], illustrates the protect effect on kidneys. Based on the above results, we found JNK and TNF-α are common contributors to kidney damage, and HMGB1 from the muscle may trigger them. Thus, the hypothetical relationship was built.

There are some limitations in this study. Firstly, apoptosis has been reported to be involved in kidney damage at the cellular level after experimental CS [25, 30]. In fact, necrosis is also involved in kidney damage [25]. It is unilateral that we choose apoptosis to detect cellular survival status. Secondly, HMGB1 induced activation of JNK mainly depends on the receptors, including toll-like family receptors and the receptor for advanced glycation end-products. Regrettably, our study does not address this point.

## Conclusions

In conclusion, it is possible that JNK and TNF-α commonly contribute to kidney damage, by assembling a positive feedback cycle after CS, leading to increased apoptosis in the renal cortex. HMGB1 from the muscle may be the trigger.

## Abbreviations

CS: Crush syndrome
HE staining: Hematoxylin-eosin staining
HMGB1: High mobility group box 1 protein
JNK: c-Jun N-terminal kinase
TNF-α: Tumor necrosis factor-α
TUNEL: Terminal deoxynucleotidyl transferase-mediated dUTP nick end-labeling
